# Valorization of *Sargassum muticum* Biomass According to the Biorefinery Concept

**DOI:** 10.3390/md13063745

**Published:** 2015-06-11

**Authors:** Elena M. Balboa, Andrés Moure, Herminia Domínguez

**Affiliations:** 1 Department of Chemical Engineering, University of Vigo (Campus Ourense), Polytechnical Building, As Lagoas s/n, Ourense 32004, Spain; E-Mails: amoure@uvigo.es (A.M.); herminia@uvigo.es (H.D.); 2 Research Transfer and Innovation Centre (CITI), University of Vigo, Tecnopole, Rúa Galicia, 2, Ourense 32900, Spain

**Keywords:** *Sargassum muticum*, biorefinery, autohydrolysis, antioxidant, SC*-*CO_2_, fucoxanthin

## Abstract

The biorefinery concept integrates processes and technologies for an efficient biomass conversion using all components of a feedstock. *Sargassum muticum* is an invasive brown algae which could be regarded as a renewable resource susceptible of individual valorization of the constituent fractions into high added-value compounds. Microwave drying technology can be proposed before conventional ethanol extraction of algal biomass, and supercritical fluid extraction with CO_2_ was useful to extract fucoxanthin and for the fractionation of crude ethanol extracts. Hydrothermal processing is proposed to fractionate the algal biomass and to solubilize the fucoidan and phlorotannin fractions. Membrane technology was proposed to concentrate these fractions and obtain salt- and arsenic-free saccharidic fractions. Based on these technologies, this study presents a multipurpose process to obtain six different products with potential applications for nutraceutical, cosmetic and pharmaceutical industries.

## 1. Introduction

*Sargassum muticum* (Yendo) Fensholt is an invasive species of brown algae in European and American West coasts originally from Japan. During the last decades, a growing interest in the study and valorization of this seaweed has been observed, because its high rate growth represents a problem for the local ecosystem, fishing and recreational activities. Current industry has already been using it for alginate extraction. In addition, *S. muticum* biomass was expected to present antioxidant activities, as it has been used for years in Traditional Chinese Medicine [1] and there are interesting studies presenting algal components and extracts, such as fucoidan, phenolics, carotenoids, sugars, lipids, minerals, and isolated compounds among others to be utilized in food, feed, cosmetic and pharmaceutical industries [2,3,4,5,6,7].

González-López *et al.* [8] have proposed an alternative fractionation of *S. muticum* based on an integral utilization of the algae. Hydrothermal treatments and SC-CO_2_ extraction provided bioactive extracts that showed radical scavenging capacity, antimicrobial and potential for cosmetic applications [3]. Autohydrolysis is an environmentally friendly process, suitable for fractionation of terrestrial [9] and macroalgal [2,8] biomass. Operation with pressurized water under subcritical conditions is a green process [10] and has advantages derived from (a) the generation of new antioxidants formed from Maillard, caramelization and thermoxidation reactions; (b) the lower water polarity, which favors the extraction of apolar components; and (c) autocatalyzed reactions that solubilize the carbohydrate fractions. Brown algae contain anionic polysaccharides, alginates or heteroglycans rich in sulfated l-fucose. Alginates are found in the brown algae as the calcium, magnesium and sodium salts of alginic acid. This polymer consists of (1,4) linked β-d-mannuronic acid (M) and α-l-guluronic acid (G) units. The extraction step can be used to control the viscosity of the final product, demanded by the food, cosmetic and pharmaceutical industries for their thickening and gel-forming abilities, which are dependent on the M/G ratios. Recent interest in the extraction and characterization of *Sargassum* sp. alginates has arisen [11,12].

The aim of this work was to propose an alternative valorization of *Sargassum muticum* biomass based on the biorefinery concept giving rise to added-value byproducts. Green extraction and fractionation processes have been proposed to obtain alginate, fucoxanthin, phlorotannin and fucoidan rich fractions with antioxidant activity.

## 2. Results and Discussion

### 2.1. Drying

#### 2.1.1. *Sargassum muticum* Biomass

**Figure 1:**
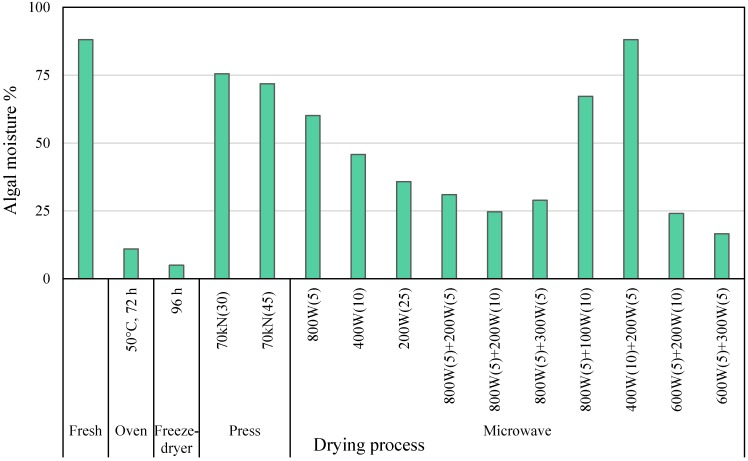
Effects of different drying technologies on the moisture content of *S. muticum* (Sm). Parenthesis are used to indicate minutes of condition treatment.

Different processes for conditioning of *S. muticum* (Sm) raw material, including pressing, and oven-, freeze- and microwave-drying were tested. The accomplished moisture reduction is shown in Figure 1. The best systems to remove water content from fresh Sm were oven- and freeze-drying, which allowed moisture reductions of 86% and 94%, respectively. For pressing and microwave-drying, the influence of pressing time, the irradiation power, and irradiation time was studied and a combination of several power and times were evaluated. Microwave-drying reached up to 83% moisture reduction when the operational conditions were two drying cycles of 5 min at 600 W and 5 min at 300 W. In addition, this process produces changes in the seaweed microstructure, such as cell rupture [13], increasing diffusivity and contact surface area, thus enhancing mass transfer and favoring molecular interactions between solvent and solutes during further extraction. Microwave-drying was a quicker alternative for Sm drying, as it was reported by Zhan *et al.* [14], who reported that microwave drying in combination with other drying methods clearly reduces food drying times. Furthermore, oven-drying was harmful to the phenolic content and antioxidant properties of extracts obtained from oven-dried *S. muticum* (ovSm) [15].

#### 2.1.2. Concentrated Extracts

Spray- and freeze-drying did not show significant influence on the composition and properties of extracts obtained from the same autohydrolysis liquor (non isothermal conditions, log R_0_ 3.79). The final extract denoted by *Product 3* dried in a freeze-dryer contained 120 ± 1.0 mg GAE g^−1^ extract, whereas the extract dried in a spray-dryer contained 113 ± 1.0 mg GAE g^−1^ extract. Radical scavenging capacity was 0.149 and 0.126 g Trolox g^−1^ extract, and EC_50_ ABTS 2.24 ± 0.01 g L^−1^ and 2.82 g L^−1^, respectively. It was observed that sample losses were obviously higher for spray drying technology but the drying time was lower than for freeze-drying (data not shown).

### 2.2. Extracts Production by Green Technologies from Sm

**Figure 2:**
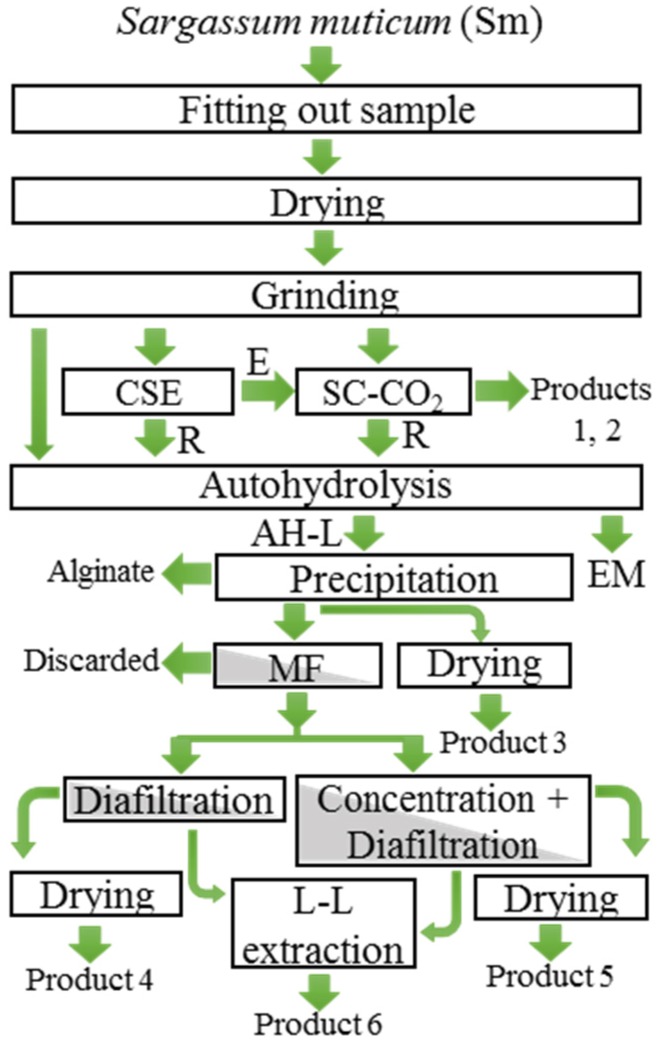
Flowchart of biorefinery processes described in this work. CSE, conventional solvent extraction with absolute and 96% ethanol; SC-CO_2_, supercritical CO_2_ extraction; MF, membrane microfiltration; L-L extraction, liquid-liquid extraction or partition; E, crude ethanolic extract; R, exhausted Sm; AH-L, Autohydrolysis liquor; EM, Exhausted autohydrolysis material; *Product 1*, fucoxanthin-enriched SC-CO_2_ fraction from E; *Product 2*, fucoxanthin-enriched SC-CO_2_ extract from Sm; *Product 3*, freeze- or spray-dried autohydrolysis liquors from ovSm; *Product 4*, diafiltrated and freeze-dried permeate from membrane microfiltrated autohydrolysis liquors; *Product 5*, spray-dried retentate from concentration of microfiltered autohydrolysis liquors; *Product 6*, pooled ethyl acetate fractions of permeates generated by previous membrane diafiltration.

This section includes the discussion of the results for the processes used under the biorefinery concept (Figure 2) to optimize the use of algal biomass [16]. Green processes were preferred, as this was considered a form of waste prevention; the use of biorenewable and less toxic solvents for humans and the environment, solvent recyclability, and shorter process times than for conventional extraction processes were also preferred.

#### 2.2.1. Conventional Solvent Extraction.

Effect of sample moisture content on the solvent extraction was evaluated. Fresh and both oven- and freeze-dried Sm samples were contacted with ethanol and/or water in a Soxhlet extractor and a stirring batch extractor. Data from Table 1 revealed that, as a general trend, sample moisture content presented a positive correlation with the solvent extraction yield. Extraction yields were six times higher from fresh samples than that from ovSm in Soxhlet extraction. Extraction yield was higher for ovSm (26.3%) than for freeze-dried *S. muticum* (fzSm) (14%) or microwave-dried *S. muticum* (mwSm) (600 W(5 min) + 200 W(10 min)) (10%), and the phenolic content was higher for extracts obtained from samples with lower moisture content (80 mg GAE g^−1^ extract). Pure ethanol extract was the most active (0.72 g Trolox g^−1^ extract).

**Table 1:** Ethanol (Et), water (W) and ethanol:H_2_O (Et:W; 3:1; 1:1) extraction yield, total phenolic content and antioxidant activity of fresh, oven (ovSm), and freeze-dried (fzSm) Sm solvent extracts (E). TPC, total phenolic content; GAE, gallic acid equivalents; TEAC, trolox equivalent antioxidant activity; SD, standard deviation.

	Fresh Sm		ovSm		fzSm			
	Soxhlet	Shaker	Soxhlet	Shaker	Shaker			
	Et		Et		W	Et	Et:W (3:1)	Et:W (1:1)
**Extraction yield (mg extract g^−1^ Sm d.b.)**	359	10	59	263	11	14	11	12
**SD**	5	6	8	4	2	1	6	7
**TPC (mg GAE g^−1^ extract)**	27	12	6	80	72	79	73	64
**SD**	3	0	0	0	0	2	8	8
**TEAC (mg trolox g^−1^ extract)**	**-**	46	3	-	309	72	88	99
**SD**	-	3	0	-	11	5	8	11

#### 2.2.2. Extracts Uses and Applications

Crude ethanolic extract (E) showed *in vitro* radical scavenging properties, 0.249 mg mL^−1^ of EC_50_ DPPH, 0.05 g Trolox g^−1^ extract, ferric reducing antioxidant power of 0.72 μM FeSO_4_·7 H_2_O and 0.48 mM ascorbic acid equivalents, reducing power of 0.17 mM ascorbic acid equivalents and a β-carotene (AAC) value of 311 in the protection from oxidation of a β-carotene/linoleic acid emulsion [3]. The topical application of crude extract (E) as cosmetic ingredient was tested with the irritability Episkin test. The treatment with this crude extract permitted a cell viability of 80% and did not cause cell irritation because it induced a low concentration of IL-1α (3.8 pg mL^−1^), comparable to the negative controls. As a cosmetic ingredient, it inhibited lipid oxidation at 93.96% in an avocado cream formulation, at 58.58% in a shower oil and at 13.90% in a massage oil stored at 50 °C for 34 days [3]. It was also studied for biological activities and it presented cytotoxicity at values of EC_50_ of 100 μg mL^−1^ on murine melanoma B16-F10 cell line [17].

#### 2.2.3. SC-CO_2_ Extraction

##### Direct Extraction

Pérez *et al.* [2] showed the effect of microwave irradiation on the cell wall structure and its destruction, thus, microwave drying was proposed as a conditioning stage before SC-CO_2_ extraction. Table 2 summarizes the information on the influence of the microwave drying conditions on the extraction yield and fucoxanthin content of the extract (*Product 2*). The SC-CO_2_ extraction tested enhanced yields and purity of the extracts, particularly those obtained at 35 MPa.

Fucoxanthin-rich extracts have been indicated as ingredients in functional foods, pharmaceutical and nutraceuticals for obesity prevention [18].

**Table 2:** Performance of SC-CO2 extracts (*Product 2*) from two different microwave-drying conditions: A, 600 W(5 min) + 300 W(5 min); B, 600 W(5 min) + 200 W(10 min). mwSm, microwave-dried *S. muticum.*

	A (17% Moisture mwSm)		B (24% Moisture mwSm)	
**SC-CO_2_ (45 °C, 1 h, 25 g CO_2_ min^−1^)**	**10 MPa**	**35 MPa**	**10 MPa**	**35 MPa**
**Extract yield (mg extract g^−1^ Sm)**	54	160	35	84
**SD**	10	10	20	10
**Fucoxanthin concentration (mg fucoxanthin g^−1^ Sm d.b.)**	5.13	0.11	2.77	0.07
**SD**	0	10	0	1

##### SC-CO_2_ Fractionation of Ethanol Extracts

In order to improve the fucoxanthin content of the ethanolic extracts, a purification protocol using SC-CO_2_ was proposed. A concentrated extract containing 12 mg commercial fucoxanthin 100 g^−1^ extract was prepared for this task (Sigma-Aldrich, St. Louis, MO, USA). SC-CO_2_ extraction was performed at three different temperatures (40, 50, 60 °C) to yield fractions (*Product* *1*) richer in fucoxanthin than the crude extract (E). Fucoxanthin content was higher when process was performed at the lowest tested temperature as seen in Table 3. The potential of low pressures to selectively remove other components in crude extracts has been used for purification purposes since they led to low fucoxanthin yields. Fucoxanthin was concentrated in the residue from the crude extracts from *Ecklonia cava* obtained using a medium chain fatty acid as co-solvent at temperatures in the range 40–50 °C and 9.7–12.4 MPa [19].

**Table 3:** Fraction yield (mg fraction g^−1^ Sm extract) and fucoxanthin content of extracts (*Product 1*) collected in vessel 1 at 35 MPa at 40, 50, 60 °C SC-CO2 extraction temperature. No extract was collected in vessel 2.

SC-CO_2_ Extraction Temperature (°C)	Fraction Yield (mg Fraction g^−1^ Sm Extract) Vessel 1	Fucoxanthin Content (mg Fucoxanthin g^−1^ Extract)
40	3	7
50	4	1
60	4	1

#### 2.2.4. Hydrothermal Treatments: Autohydrolysis or Subcritical Water Extraction

Fresh and oven-dried (ovSm) Sm were subjected to hydrolytic treatments (log R_0_ 3.46) to release alginate fraction and subsequent extract recovering as it was described above. These liquors were compared with regard to yield and properties. Extract yield was 250 mg and 260 mg extract g^−1^ Sm d.b., total phenolic content was 0.11 and 0.091 (g GAE g^−1^ extract), and TEAC was 2.16 and 1.92 g Trolox g^−^^1^ extract, for fresh and ovSm autohydrolysis liquors, respectively. EC_50_ DPPH was 1.37 for liquor obtained from fresh Sm. Sugar content for autohydrolysis liquor obtained from ovSm was 10.23%, in a proportion of fucose (1):galactose (0.96):xylose (0.71):glucose (0.65):mannose (0.06).

The autohydrolysis extraction yield (log R_0_ 4.039) from ovSm was 320 mg extract g^−1^ Sm and total phenolic content was 98 mg GAE g^−1^ extract and 41 mg Phl g^−1^ extract.

##### Membrane Processing of Alginate Free Liquors

The addition of 1% CaCl_2_ and further centrifugation provoked an increase in the salt concentration on alginate-free liquors because of the excess of CaCl_2_ not involved in the residual alginate precipitation. These liquors were treated to produce natural extracts with bioactive properties using membrane technology. Two different protocols were assayed, a sequential discontinuous diafiltration stage for impurity and salt removal and a combined process of concentration and further diafiltration.

Concentration operating in the 0.3 kDa membrane led to a solids concentration of 3 g L^−1^ in the retentate, presenting 60 mg GAE g^−1^ extract. Furthermore, when the autohydrolysis liquor was directly subjected to 1% CaCl_2_ addition solids concentration were 6 g L^−1^, and phenolic content was 151 mg GAE g^−1^ extract and 77 mg Phl g^−1^ extract. The addition of ethanol led to a slight decrease in the dried weight (from 11.73 to 9.79 g L^−1^) and in the phenolic content of the liquors (84 mg GAE g^−1^ extract).

##### Purification of AH Liquors and Arsenic Removal by Membranes

The diafiltration process was carried out to remove salt and inorganic arsenic from the alginate-free liquors using a 1 kDa cut-off membrane. Table 4 shows the successive desalting effect observed during diafiltration expressed as a concentration (g CaCl_2_ equivalents L^−1^) of alginate-free autohydrolysis liquors obtained from ovSm. The initial salt concentration value (2.99 g CaCl_2_E L^−1^) in the autohydrolysis liquors increased four times owing to the alginate precipitation with 1% CaCl_2_. Because a high salt concentration is not desirable for the antioxidant extract formulation, its removal in a three-stage discontinuous diafiltration process was carried out. Salt concentration was reduced down to 0.24 g CaCl_2_E L^−1^, as shown in process (i). Process (ii) data of salt concentration and phenolic content is also shown. The product obtained from concentration on 1 kDa membrane presented 32.95 g CaCl_2_E L^−1^, similar to the permeate (30.36 g CaCl_2_E L^−1^). Salt concentration decreased progressively after each diafiltration down to 2.23 g salt L^−1^ after the seventh diafiltration, whereas the phenolic content was 0.09 g GAE g^−1^ extract. *Product 5* yielded 0.246 g g^−1^ Sm after spray-drying. *Products 4* and *5* were investigated for ash content and values of 1.9 ± 0.35 and 5.68 ± 0.28 g ash 100 g^−1^ product were found, respectively.

**Figure 3:**
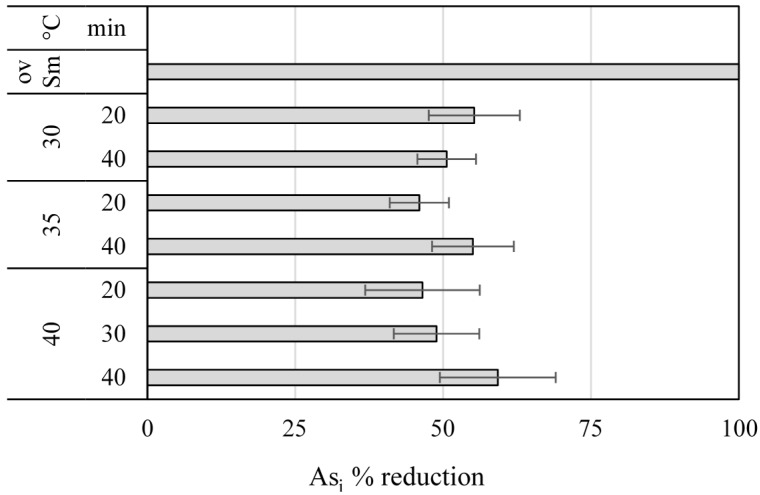
Inorganic arsenic (As_i_) % reduction of soaked Sm compared with ovSm.

Salt elimination involves ash content and arsenic (As) reduction. Due to the toxicological importance of this heavy metal, further analysis of As content in the final products was addressed. Different strategies for As removal have been tried based on information from the literature with the aim of obtaining arsenic free products, including: (a) extraction with ethanol at different concentrations; (b) aqueous extraction at different time and temperature [20,21]; (c) ultrasound assisted ethanol extraction; (d) accelerated solvent extraction using ethanol [22]; (e) adsorption onto orange peel as sorbent [23]; (f) adsorption onto activated charcoal; and (g) ultrafiltration membrane fractionation. Strategies a, c, d, e, and f did not allow the As rate to decrease, but interesting results were observed for aqueous soaking at 35 °C for 20 min; a reduction of 45.99% ± 5.0% of As was observed in Sm (Figure 3). Significant reduction was also observed for membrane fractionation of Sm extracts. Comparative data of the arsenic removal with these techniques is shown in Table 5: Inorganic arsenic (As_i_) content of an autohydrolysis liquor from Sm from June 2011 (3.46 log R_0_, 3.75 L volume Parr**)**, the spray-dried liquors (*Products 4* and *5*) and the retentate and permeate streams from the 1 kDa cut off spiral membrane. Membrane processing allowed a selective removal of these species, since the concentrate showed a reduction of As_i_ of 90%, whereas permeate (18%), *Product 4* (96%) and *Product 5* (99%) compared to the autohydrolysis extract values. The remaining As in the concentrated product was probably biologically unavailable, since no toxicity was detected in an oral study. Muñoz *et al.* [24] reported that inorganic forms of As are the most toxic and total As measurement is not a good pointer to report a tolerable daily intake. Among As_i_, the trivalent arsenicals are more toxic than the pentavalent arsenicals [25].

**Table 4:** (***i***) Desalting effect observed in the three steps diafiltration process of Liquor + CaCl2 from ovSm (3.16 log R0) with a 1 kDa Amicon membrane; (***ii***) Desalting effect observed in one step concentration and seven steps diafiltration process of microfiltrated autohydrolysis liquor from fresh Sm (3.46 log R0) with a 1 kDa Amicon membrane. CaCl2E, CaCl2 equivalents; GAE, gallic acid equivalents; Ret, retentate; Perm, permeate.

(*i*) Stream	Calculated Salt Concentration (g CaCl_2_E L^−1^)	(*ii*) Stream	Calculated Salt Concentration (g CaCl_2_E L^−1^)	Phenolic Content (g GAE g^−1^ Extract)
Liquor + 1% CaCl_2_	12.49	**Concentrate**	32.95	0.05
Ret 1	5.58	**Perm**	30.36	0.03
Perm 1	5.38	**Ret 1 ׀ Perm 1**	19.27 ׀ 19.98	- ׀ 0.03
Ret 2	1.01	**Ret 2 ׀ Perm 2**	11.87 ׀ 11.42	0.14 ׀ 0.02
Perm 2	0.71	**Ret 3 ׀ Perm 3**	- ׀ 6.60	0.08 ׀ 0.03
Ret 3	0.24	**Ret 4 ׀ Perm 4**	5.53 ׀ 4.46	0.08 ׀ 0.03
Perm 3	0.20	**Ret 5 ׀ Perm 5**	4.28 ׀ 2.85	0.08 ׀ 0.03
Pooled Perm	1.81	**Ret 6 ׀ Perm 6**	3.12 ׀ 1.56	0.09 ׀ 0.03
		**Ret 7 ׀ Perm 7**	2.23 ׀ 0.98	0.09 ׀ 0.02

**Table 5:** Inorganic arsenic (Asi) values of autohydrolysis liquor, *Product 4*, *Product 5* and retentate and permeate streams from the 1 kDa membrane. SD, standard deviation.

	As_i_ μg g^−1^ ± SD
Autohydrolysis liquor	29.4 ± 2.9
1 kDa retentate	2.8 ± 0.6
1 kDa permeate	24.1 ± 2.0
*Product 4*	1.1 ± 0.3
*Product 5*	0.4 ± 0.1

##### Extracts Uses and Applications

The spray-dried membrane concentrated liquors from autohydrolysis (*Products 4* and *5*) were nontoxic when administered orally to Wistar rats. Furthermore, increased levels of antioxidant hepatic enzymes were observed [26].

The solid residue remaining after Sm autohydrolysis contains minerals [8] and could be suited for fertilization purposes and reparation of soil amendments, since the composition was similar to that of other brown algal fertilizers. Aqueous extracts of brown algae, such as *Sargassum wighti* and *Caulerpa chemnitzia*, performed better when compared to the water soaked controls on growth and biochemical constituents of *Vigna sinensis* [27]. The application of brown seaweeds (*Spatoglossum asperum* and *Sargassum swartzii*) as soil amendment showed a significant suppressive effect on the root-rotting fungi *Macrophomina phaseolina*, *Rhizoctonia solani* and *Fusarium solani,* and on the root-knot nematode *Meloidogyne javanica* in tomato roots [28]. *Product 6* from solvent partition was proposed as phenolic rich extract for cosmetic formulations.

## 3. Materials and Methods

### 3.1. Fitting Out Sample

*Sargassum muticum* (Sm) was collected in Praia da Mourisca (Pontevedra, Spain) in summer 2010, 2011, 2012 and in December 2011. The algal material was cleaned from epiphytes and sand under running tap water, drained and further stored in sealed plastic bags or glass bottles at −18 °C until use. Defrosting was performed at room temperature, letting excess water drain out. In some cases, Sm was not frozen and was freshly milled for direct utilization.

### 3.2. Drying of Sm and Extracts

*Pressing.* The free water content of fresh Sm was removed with a pneumatic press (P142, Enerpac, Milwaukee, WI, USA) working at 70 kN for 30 or 45 min.

*Oven-drying.* Fresh Sm was manually distributed among paper trays and afterward placed in a convection lab oven at 50 °C for 72 h.

*Freeze-drying.* Frozen Sm and samples from the different processes assayed were dried in a freeze dryer (Alpha 1-4 LSC, Martin Christ, Germany) operating during 96 h at −80 °C and 0.0010 mbar.

*Microwave-drying.* Defrosted *Sm* was dried in a solvent-free microwave/gravity extraction system (NEOS-GR, Milestone srl, Milan, Italy). Several power-temperature profile and operational time were assayed to determine the optimal drying variable conditions and their influence in the later process.

*Spray-drying of extracts.* A mini spray dryer (B-290, Büchi, Flawil, Switzerland) was used.

### 3.3. Grinding

Dried Sm sample was ground in a universal mill (M20, IKA, Staufen, Germany) until a particle size of less than 1 mm. A classic kitchen chopper (Moulinex, Barcelona, Spain) was used for fresh Sm material grinding.

### 3.4. Proposal of Global Valorization of Sm for the Recovery of Alginate, Fucoxanthin and Antioxidant Compounds

In order to achieve an optimal approach to a better use of Sm biomass, a process with several variants was proposed. Different kinds of drying techniques were tested and their influence on the target compound yields was evaluated. Green technologies were combined to propose a sequential extraction of the different target compounds studied. Since a recent work studied the environmental assessment of some Sm valorization strategies [29], this study focused on the selection of optimized processes. The studied alternatives and their products are shown in Figure 2. Microwave- and freeze-dried Sm were subjected to two different independent processes: (a) absolute and 96% ethanol extraction to obtain a crude ethanolic extract (E) and the exhausted Sm (R). Fresh Sm was also utilized for (a). E was further fractionated by SC-CO_2_ (*Product 1*). (b) SC-CO_2_ extraction to yield fractions (*Product 2*) and exhausted Sm (R); R can be used for autohydrolysis extraction. Furthermore, fresh and oven-dried Sm (ovSm) were subjected to autohydrolysis. Solid exhausted material (EM) was separated by filtration and CaCl_2_ was added for alginate precipitation. Autohydrolysis liquors (AH-L) from ovSm were freeze- or spray-dried to yield *Product 3*. Finally, autohydrolysis liquors from fresh Sm was in turn passed through a microfiltration membrane. Retentate was discarded but permeate was used for obtaining two products: First, liquor was diafiltrated and freeze-dried to obtain *Product 4*. Later, another batch of liquor was microfiltrated, concentrated and further spray-dried to get *Product 5*. Permeates were partitioned with ethyl acetate and pooled to yield *Product 6*.

#### 3.4.1. Conventional Solvent Extraction

Solvent extraction was carried out using different ethanol concentration owing to the different target compounds. Crude ethanolic extract (E) production was accomplished with 96% ethanol at a liquid-solid ratio (LSR) of 10 at 40 °C for 3 h, in the absence of light under rotary agitation in an orbital shaker. Fucoxanthin enriched extracts were obtained after contacting 10 mL of absolute ethanol with 1 g of ground dry alga. Extraction was composed of a two-stage process carried out in darkness, at 40 °C during 6 h. An extraction control was done with Sm overnight in Soxhlet with 96% ethanol at a LSR of 20. Solids were separated by filtration through Whatman No. 1 filter paper and liquors were filtered and evaporated at 40 °C under vacuum in a Rotavapor system (R-114, Büchi, Switzerland).

#### 3.4.2. SC-CO_2_ Extraction

A pilot plant SF extractor (SFE-1000F-2-C10, Thar Technologies Inc., Pittsburgh, PA, USA) with a 1 L cylinder extractor and two 0.5 L separators was used.

Fucoxanthin concentration enhancement from crude ethanolic extracts (E) was proposed using different sample pretreatments to evaluate the drying effect on the extract yields of *Product 1*. The raffinates (R) were susceptible to be subjected to autohydrolysis treatment.

The SC-CO_2_ extraction was further applied to microwave-dried samples. Microwave-dried Sm biomass (20 g) was placed into the extractor and packed with glass beads in a cloth to avoid solids from letting off the extractor and blocking the circuit. CO_2_ was cooled with a circulating bath (model 9506, PolyScience, Niles, IL, USA) and pumped at a flow rate of 25 g CO_2_ min^−1^ by a piston pump (P-200A, Thar Technologies, Inc., Pittsburgh, PA, USA). The extraction process lasted 1 h and the extract was collected in the first separator. Operating temperature was 45 °C and pressure was set at 10 and 35 MPa, respectively, in two extractions ran in duplicate. Extracts were collected and the separator was washed with ethanol and combined with the extract before the ethanol evaporation in a rotavapor system. Extracts were stored at −20 °C under N_2_ until analysis. These extracts were denoted *Product 2*.

#### 3.4.3. Fucoxanthin Concentration Enhancement by SC-CO_2_ Fractionation

Five grams of crude ethanolic extract (E) were extracted at 35 MPa during 1 h with a CO_2_ flux of 25 g CO_2_ min^−1^. Extraction was carried out in duplicate. The first separator was set at 4 MPa, and the second one, to 1 MPa. Operating temperature was 40, 50 and 60 °C in three different processes.

#### 3.4.4. Autohydrolysis or Subcritical Water Extraction

Autohydrolysis treatments were performed in three different stainless steel reactors (model 4842 (0.6 L volume), model 4848 (3.75 L) and model 4843 (19 L), Parr Instrument Co., Moline, IL, USA). Processes were run in non-isothermal conditions and the variables were LSR, temperature and agitation speed. Severity factor (R_0_) was calculated by the equation: log R_0_ = log [R_0__heating_ + R_0__cooling_] = log [ʃ_0_^tmax^(T(t) − T_Ref_) × dt/ω + ʃ^tf^_tmax_(T′ − T_Ref_) × dt/ω]; where T, temperature; T_ref_ reference temperature (100 °C); ω, activation energy of reaction (14.75); *t*, reaction time, reported by Romaní *et al.* [30], and expressed as dimensionless. Time was standardized since the heating profile was different.

The effect of drying on extraction yield was evaluated subjecting fresh and ovSm to an autohydrolysis extraction. Process was carried out by contacting 30 g water with 1 g Sm d.b. and heated in the reactor until reaching log R_0_ in the range of 2.52–4.43. Once the target temperature was reached, the reactor was cooled immediately and solid phases were separated by filtration. A part of liquors was subjected to further fractionation and other was dried to obtain *Product 3*. For selected log R_0_ process was scaled up to obtain higher liquor volumes.

#### 3.4.5. Alginate Precipitation

Alginate precipitation was carried out after autohydrolysis by adding ethanol and/or 1% CaCl_2_ to the liquor and stirring overnight at room temperature. This is a post-autohydrolysis alginate precipitation in opposition to the autohydrolysis pretreatment of algal biomass reported by González-López *et al.* [8] yielding 10.1% alginate.

#### 3.4.6. Fractionation by Means of Membrane Technology

Membrane technology was used to concentrate the liquors obtained after the alginate precipitation, followed by a diafiltration stage to yield *Products 4* and *5*. It was also used to remove salt and arsenic from the alginate free liquors. In order to obtain *Product 4*, autohydrolysis liquors were subjected to microfiltration and diafiltration in a 200 Da membrane. Salt removal was accomplished in 6 steps. *Product 5* was obtained by concentrating microfiltered liquors by a 1 kDa membrane and further diafiltration in the same membrane. Acceptable levels of salt and As were achieved after 7 steps.

Concentration experiments were carried out in batch mode, with simultaneous retentate recycling and permeate removal. Membrane operation was performed in a homemade filtration pilot plant, consisting of a 10 L feed tank, a variable speed pump (Hydracell, Minneapolis, MN, USA) and a membrane module. Pressure was monitored at the entrance and exit of the membrane module, and a needle valve located after it was used for transmembrane pressure (TMP) regulation. Temperature was monitored with a PT100 probe, and controlled by flushing tap water through a refrigeration coil placed in the feed tank. The pilot plant was equipped with different membranes, according to the purpose chosen. The commercial micro-, ultra- and nanofiltration membranes used in this work are described below.

*Microfiltration* was carried out in a polymeric spiral membrane with the following characteristics: 60.7 cm diameter, 1.016 m length, 0.77 m effective area, 0.1 μm pore size, 6 L m^−2^ h^−1^ water permeability, supplied by Iberlact (Alcala de Henares, Spain).

*Ultrafiltration* was performed in a Prep/Scale TFF 6 ft^2^ Cartridge, vertical, spiral, made of regenerated cellulose with the following characteristics: 1 kDa MWCO cut-off, 5.8 cm diameter, 39.9 cm length, 14 L m^−2^ h^−1^ water permeability supplied by Millipore (Billerica, MA, USA).

*Diafiltration* process was performed with three different membrane setups: (a) through a 200 Da polymeric spiral membrane (42 L m^−2^ h^−1^ water permeability); (b) the same 1 kDa Prep/Scale TFF 6 ft^2^ Cartridge used for concentration and (c) a 400 mL stirred cell series 8000 Amicon (Millipore, Billerica, MA, USA) with 0.3 and 1 kDa membranes.

Conductivity (mS) was recorded to monitor the process and a CaCl_2_ calibration curve (*y* = 1.1209*x*) was used to calculate the salt concentrations (CaCl_2_ equivalents L^−1^).

#### 3.4.7. Fractionation by Means of Immiscible Solvents (Partition)

Some permeates from diafiltration processes were further liquid-liquid extracted by adding ethylacetate (EA) in a permeate:EA volume ratio (1:3) by stirring at room temperature and decanting the mixture. EA fraction was evaporated under vacuum in a rotavapor system to obtain *Product 6* and kept at −20 °C until analysis.

### 3.5. Analytical Methods

#### 3.5.1. Non-Volatile Compounds Yield

Extraction yield was calculated gravimetrically by weighting an amount of sample before and after letting it dry for 24 h at 105 °C, and expressed as g extract g^−1^ Sm d.b. or percentage (%) of Sm d.b.

#### 3.5.2. Inorganic Arsenic Determination

Inorganic arsenic determination was performed by the Department of Analytical Chemistry, Nutrition and Bromatology, Faculty of Chemistry (University of Santiago de Compostela, Spain). Briefly, sample was extracted with water and ammonium bicarbonate in an ultrasound water bath, and inorganic arsenic quantification was made before separation of arsenic species by HPLC and further determination by ICP-MS. Shown data are the sum of As_III_ and As_V_ species. Tests were run in triplicate.

#### 3.5.3. Total Phenolic Content (TPC) and Antioxidant Activity

TPC was colorimetrically determined as in Singleton and Rossi [31] using the Folin-Ciocalteu reagent (Sigma-Aldrich, St. Louis, MO, USA) and expressed as gallic acid (Sigma-Aldrich, St. Louis, MO, USA) equivalents (GAE). To express the results as g phloroglucinol (Sigma-Aldrich, St. Louis, MO, USA) equivalents (PhlE), a method described by de Quirós *et al.* [32] was used.

ABTS radical cation (ABTS^•+^) [2,2′-azinobis (3-ethylbenzothiazoline-6-sulfonic acid) diammonium salt] (Sigma-Aldrich, St. Louis, MO, USA) scavenging capacity was determined as previously described by Re *et al.* [33] and expressed as Trolox (Fluka, parent company of Sigma-Aldrich, St. Louis, MO, USA) and equivalents (TEAC, Trolox Equivalent Antioxidant Capacity). EC_50_ ABTS was defined as the extract concentration needed to inhibit 50% of the ABTS radical.

EC_50_ DPPH was defined as the extract concentration needed to inhibit 50% of the DPPH radical. DPPH (α,α-biphenyl-β-picrylhydrazyl) radical scavenging activity was determined as by von Gadow *et al.* [34].

All analyses were performed in triplicate and results are reported on a dry matter basis.

#### 3.5.4. HPLC Analysis

Fucoxanthin content of extracts was determined by reversed phase HPLC in an Agilent 1100 instrument (Waldbronn, Germany) equipped with a diode-array detector (DAD) and an ODS-2 column (5 μm, 250 mm × 4.6 mm, Spherisorb, Waters, Milford, MA, USA). Acetonitrile:methanol:water (6:2.5:1.5, v:v:v) was used as mobile phase at a flow rate of 0.8 mL min^−1^. A commercial fucoxanthin standard (Sigma-Aldrich, St. Louis, MO, USA) was used for quantification.

Sugar content was determined as described in a previous work [35].

### 3.6. Statistical Analysis

Experiments results are expressed as the average of the data and the standard deviation (SD).

## 4. Conclusions

The valorization of the invasive alga *Sargassum muticum* was proposed following different schemes of processing under a biorefinery point of view. The utilization of the carotenoid, phenolic and fucoidan fractions was addressed preferably using biorenewable, reusable and less toxic solvents (subcritical water, ethanol, ethylacetate and supercritical CO_2_) than those used for traditional processes, and green processes as autohydrolysis and SC-CO_2_ extraction and fractionation. In addition to alginate, six products were obtained, which could alternatively be proposed for food, feed, agricultural, pharmaceutical, nutraceutical and cosmetic applications.
